# Suicide Mortality During the Perinatal Period

**DOI:** 10.1001/jamanetworkopen.2024.18887

**Published:** 2024-06-27

**Authors:** Kara Zivin, Chuwen Zhong, Alejandro Rodríguez-Putnam, Emma Spring, Qingyi Cai, Alyson Miller, Lily Johns, Viktoryia A. Kalesnikava, Anna Courant, Briana Mezuk

**Affiliations:** 1 Department of Psychiatry, Michigan Medicine, Ann Arbor; 2 Department of Obstetrics and Gynecology, Michigan Medicine, Ann Arbor; 3 Center for Clinical Management Research, Veterans Affairs Ann Arbor Healthcare System, Ann Arbor, Michigan; 4 Department of Epidemiology, University of Michigan School of Public Health, Ann Arbor; 5 Research Center for Group Dynamics, Institute for Social Research, University of Michigan, Ann Arbor

## Abstract

**Importance:**

The US has the highest maternal mortality rate among developed countries. The Centers for Disease Control and Prevention deems nearly all of these deaths preventable, especially those attributable to mental health conditions. Coordination between US health care and social service systems could help further characterize circumstances and risks associated with perinatal suicide mortality.

**Objective:**

To examine contextual and individual precipitating circumstances and risks associated with perinatal suicide.

**Design, Setting, and Participants:**

This cross-sectional observational study used a convergent mixed methods design to explore factors contributing to maternal suicides and deaths of undetermined intent (hereinafter, undetermined deaths) identified in National Violent Death Reporting System (NVDRS) data for January 1, 2003, to December 31, 2021. Analyses included decedents who were aged 10 to 50 years and pregnant or post partum at death (collectively, the perinatal group) and demographically matched female decedents who were not pregnant or recently pregnant (nonperinatal group) at death. Analyses were performed between December 2022 and December 2023.

**Exposures:**

Pregnancy status at death (perinatal or nonperinatal).

**Main Outcomes and Measures:**

The main outcomes included contributing circumstances associated with suicides and undetermined deaths cited in coroner, medical examiner, or law enforcement case narratives. The study examined quantitative differences between groups using a matched analysis and characterized key themes of salient suicide circumstances using qualitative content analysis.

**Results:**

This study included 1150 perinatal decedents identified in the NVDRS: 456 (39.6%) were pregnant at death, 203 (17.7%) were pregnant within 42 days of death, and 491 (42.7%) were pregnant within 43 to 365 days before death, yielding 694 postpartum decedents. The nonperinatal comparison group included 17 655 female decedents aged 10 to 50 years. The mean (SD) age was 29.1 (7.4) years for perinatal decedents and 35.8 (10.8) years for nonperinatal decedents. Compared with matched nonperinatal decedents, perinatal decedents had higher odds of the following identified contributing circumstances: intimate partner problems (IPPs) (odds ratio [OR], 1.45 [95% CI, 1.23-1.72]), recent argument (OR, 1.33 [95% CI, 1.09-1.61]), depressed mood (OR, 1.39 [95% CI, 1.19-1.63]), substance abuse or other abuse (OR, 1.21 [95% CI, 1.03-1.42]), physical health problems (OR, 1.37 [95% CI, 1.09-1.72]), and death of a family member or friend (OR, 1.47 [95% CI, 1.06-2.02]). The findings of the qualitative analysis emphasized the importance of mental health and identified 128 decedents (12.4%) with postpartum depression.

**Conclusions and Relevance:**

This study provides insights into complex factors surrounding maternal suicide, and it highlights opportunities for further research to understand long-term consequences of perinatal mental health. These findings also underscore the need for targeted evidence-based interventions and effective policies targeting mental health, substance use, and IPPs to prevent maternal suicide and enhance maternal health outcomes.

## Key Points

**Question:**

What circumstances are associated with perinatal (ie, pregnant and postpartum) suicide, and how do they vary across the perinatal period?

**Findings:**

In this cross-sectional study using data from the National Violent Death Reporting System, perinatal decedents were more likely to experience intimate partner problems (IPPs), depressed mood, substance abuse, physical health problems, and recent bereavement compared with matched nonperinatal decedents. Qualitative analysis identified precipitating mental health circumstances, including postpartum depression.

**Meaning:**

These findings highlight the need for policy making that targets mental health, substance use, and IPPs to mitigate perinatal suicide risk.

## Teaser

This cross-sectional study uses data from the National Violent Death Reporting System to evaluate contextual and precipitating circumstances and risks associated with perinatal suicide.

## Introduction

Suicide during the perinatal period (eg, during pregnancy and ≤1 year post partum) is a leading cause of maternal mortality in the US, contributing to 8.4% of pregnancy-related deaths.^1,2^ A Centers for Disease Control and Prevention (CDC) report from 36 state maternal mortality review committees considered more than 80% of maternal deaths preventable, including 23% of deaths from mental health conditions, primarily suicide and overdoses related to substance use disorders.^2^

The US uses the World Health Organization (WHO) definition of maternal mortality as deaths of women while pregnant or within 42 days of pregnancy, from any cause related to, or aggravated by, the pregnancy or its management, but not from accidental or incidental causes.^3^ The WHO classification of deaths involving pregnancy and childbirth excludes suicides, as well as accidents and homicides, and deaths occurring within 43 to 365 days of delivery.^3^ The WHO definition thus underestimates the true burden of lives lost. Additionally, racial and ethnic disparities in maternal health—including pregnancy-related mortality—persist,^4^ as do disparities in access to mental health services across screening, diagnosis, and treatment.^5^

In the context of the White House Blueprint for Addressing the Maternal Health Crisis, which aims to address maternal mental health and reduce preventable pregnancy-related deaths,^6^ this study uses data from the National Violent Death Reporting System (NVDRS) to examine precipitating circumstances of suicide among pregnant, postpartum, and similarly aged nonpregnant women.^7^ The NVDRS is a CDC-funded, state-based surveillance system that collects data on violent deaths abstracted from investigative reports. The NVDRS is the largest collection of suicide data in the US and provides an opportunity for population-level mixed methods research.

Previous studies that have examined quantitative factors associated with pregnancy-related deaths^8,9,10,11^ have reported that mental health problems, substance use disorders, and intimate partner problems (IPPs) preceded suicide and homicide in pregnancy. Limitations of the aforementioned studies include sole use of older quantitative data sources, which typically reflect a limited number of states, smaller sample sizes, or both.

This cross-sectional observational and qualitative study had the following goals: (1) to identify characteristics more prevalent among perinatal suicide decedents compared with nonperinatal decedents, (2) to leverage quantitative and qualitative NVDRS data to explore circumstances that distinguish pregnancy and postpartum periods among perinatal decedents, and (3) to characterize key themes from circumstances among perinatal decedents. We also sought to illuminate underlying factors for perinatal suicide to inform targeted prevention strategies for this population.

## Methods

### Data Source

This cross-sectional study used data from the NVDRS for January 1, 2003, to December 31, 2021. The NVDRS captures 50 states, the District of Columbia, and Puerto Rico; however, states’ contributions vary by year.^12^ To reflect potential misclassification of suicide, we included suicides and deaths of undetermined intent (hereinafter, undetermined deaths) in the sample.^13^ The NVDRS identified decedent cases using *International Classification of Diseases, Tenth Revision, Clinical Modification* codes for suicide (X60-X84, Y87.0, and U03) and undetermined death (Y10-Y34, Y87.2, and Y89.9). The NVDRS Restricted Access Database contains text narratives that are typically between 150 and 300 words and are written by NVDRS staff using coroner, medical examiner, and law enforcement reports, death certificates, suicide notes, and interviews with decedents’ family and friends.

The University of Michigan Institutional Review Board deemed this study exempt from review and waived the need for informed consent due to the use of decedent data. We preregistered the study on the Open Science Framework^14^ and followed the Strengthening the Reporting of Observational Studies in Epidemiology (STROBE) reporting guideline.

### Quantitative Measures and Analysis

#### Exposures

According to the NVDRS pregnancy status variable, we created 2 comparisons among reproductive-aged individuals (aged 10-50 years). For the first comparison, we defined *pregnant* using the “pregnant at time of death” variable. We defined *post partum* by grouping the variables “not pregnant but pregnant within 42 days of death” and “not pregnant but pregnant within 43 days to 1 year before death.” For the second comparison, we defined *perinatal* by combining the variables “pregnant” and “postpartum.” Finally, we defined *nonperinatal* by grouping the variables “not pregnant, not otherwise specified” and “not pregnant within the last year.”

#### Outcomes

Informed by the life-course theory of suicide,^15^ we used 13 salient circumstance binary variables (yes vs no, not available, or unknown) derived from NVDRS code as outcomes. We further classified these variables into the following 6 groups: relationship problems (intimate partner, family relationship, or argument), mental illness (depression diagnosis, ≥2 diagnoses, depressed mood, current treatment for mental illness, or history of treatment for mental illness), substance problems (alcohol problem or substance or other abuse), physical health problems, job or financial problems, and death of a friend and family member. eAppendix 1 in Supplement 1 provides additional details for each circumstance.

#### Covariates

Covariates included age (grouped by quartile; 10-23, 24-28, 29-34, or 35-50 years for pregnant vs postpartum; and 10-27, 28-37, 38-44, or 45-50 years for nonperinatal vs perinatal), race and ethnicity, educational attainment (grade 12 or less, high school diploma or GED, some college or college graduate or above, or unknown), and marital status (single or never married, married or domestic partnership, or divorced, separated, or widowed). We included race and ethnicity to describe differences in suicide rates by racial and ethnic group as well as racial disparities in perinatal outcomes. These data were defined by the NVDRS and included Black, Hispanic or Latino, White, or other race or ethnicity (ie, American Indian or Alaska Native, Asian, Native Hawaiian or Pacific Islander, or unspecified race or ethnicity). We included 3 binary (yes vs no) variables related to death circumstances: injury location (ie, injured at home), autopsy (ie, whether the decedent underwent an autopsy), and in the labor force.

### Statistical Analysis

#### Quantitative Analysis

We estimated odds ratios (ORs) with 95% CIs to compare sociodemographic characteristics in 2 ways: pregnant vs postpartum and perinatal vs nonperinatal. Substantial differences in demographic characteristics between the 2 comparison groups could bias the effect of pregnancy status on circumstances. Therefore, we estimated propensity scores based on race and ethnicity, educational attainment, age group, and state, using optimal full matching to generate matched datasets for the 2 comparison groups, respectively (eMethods in Supplement 1). After matching, we used 2 sets of logistic regression models with robust SEs to calculate associations between pregnancy status (perinatal vs nonpregnant and postpartum vs pregnant) and circumstances surrounding suicide.^16^ The models controlled for the aforementioned sociodemographic characteristics. *P* < .05 (2-tailed) was considered statistically significant. We ran 2 post hoc sensitivity analyses to examine the robustness of our findings. First, we examined the association between cause of death and pregnancy status. Then we repeated the matching process and logistic regressions analyses, excluding undetermined deaths.

#### Qualitative Measures and Analysis

Using thematic analysis, we employed an iterative, multistep approach to analyze qualitative narrative data based on grounded theory and open coding procedures (eAppendices 2 and 3 in Supplement 1).^17,18,19^ Initially, 2 reviewers (E.S. and A.M.) annotated 200 of the narratives for familiarity. Five annotators (C.Z., A.R.-P., E.S., Q.C., and A.M.) collaboratively conducted a second manual exploratory review of 550 of the narratives to generate or add complex themes and capture more granular information related to the timing of circumstances in the perinatal period.

Based on the life-course theory of suicide^15^ and building on circumstantial variables, we created a hierarchical codebook with 12 major themes (representing 48 subthemes), organized within 3 overarching domains: (1) relationship conflicts, (2) health history, and (3) contextual and social or environmental factors and other circumstances. eAppendix 2 in Supplement 1 provides details of themes, subthemes, and definitions used in qualitative coding.

Using the codebook, 6 annotators (C.Z., A.R.-P., E.S., Q.C., A.M., and L.J.) independently annotated all 1032 qualitative narratives. Interannotator agreement^20^ across the main themes was strong (α = 0.84). The Potato open-source annotation tool facilitated qualitative annotations and analysis.^12^

We conducted statistical analyses using SAS software, version 9.4 (SAS Institute Inc), and RStudio, version 2023.06.2+561 (R Project for Statistical Computing). Analyses were performed between December 2022 and December 2023.

## Results

### Cohort Characteristics

**Figure 1.**
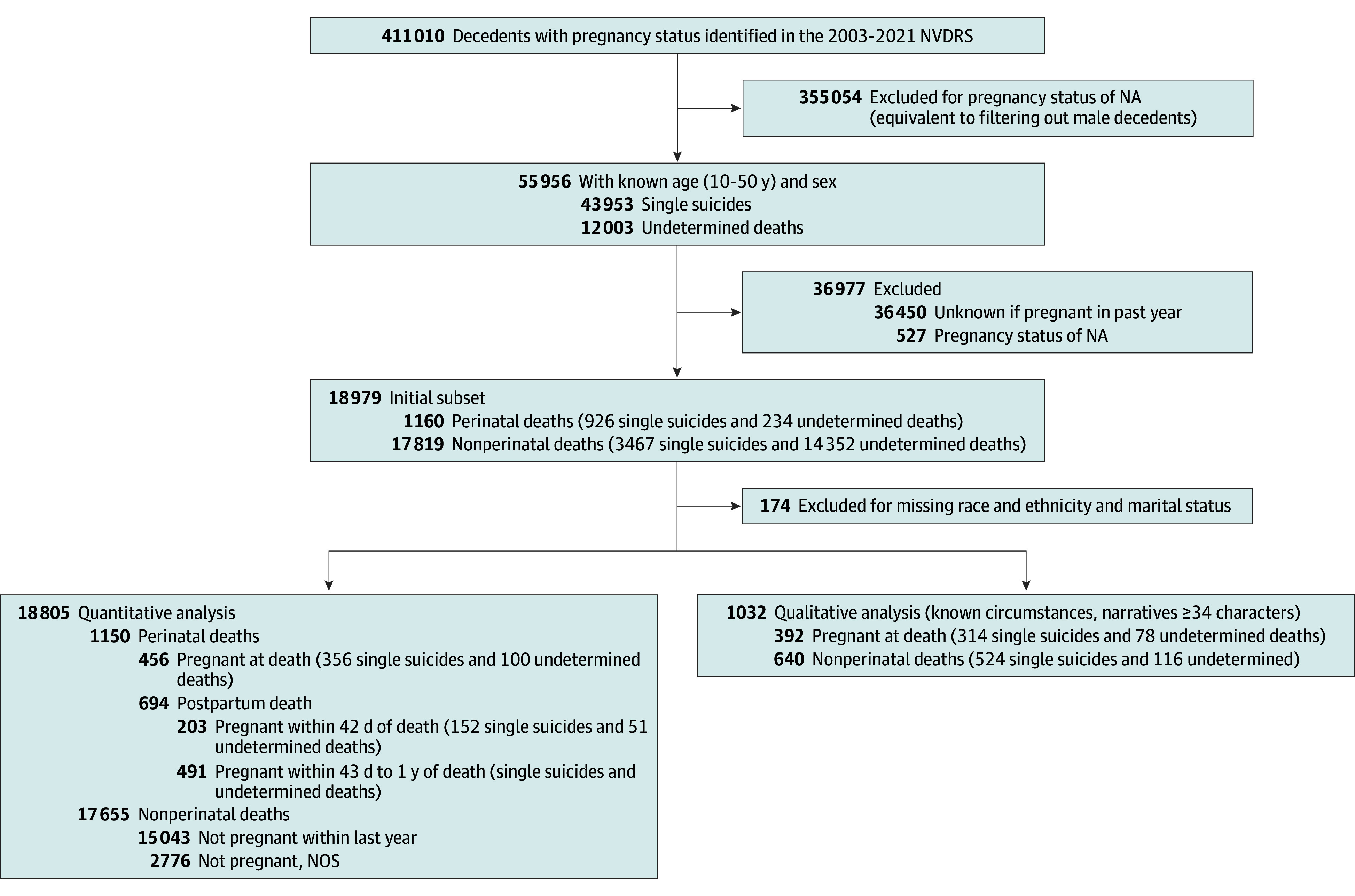
Study Flowchart The National Violent Death Reporting System (NVDRS) pregnancy category “pregnant, not otherwise specified” (NOS) (n = 42) was evaluated via manual review of text narratives. The following were included in the counts, when applicable: current pregnancy (n = 5), no mention of recent or current pregnancy (n = 26), not pregnant but pregnant 43 days to 1 year before death (n = 7), not pregnant but pregnant within 42 days of death (n = 2), and recent pregnancy (timing unknown) (n = 2). NA indicates not applicable.

Figure 1 describes the sample selection and study cohorts, and the eFigure in Supplement 1 describes the study design. In the 2003-2021 NVDRS data, we initially found 1160 perinatal decedents (926 suicides and 234 undetermined deaths) and 17 819 nonperinatal decedents (3467 suicides and 14 352 undetermined deaths); we then excluded 10 decedents from the perinatal group and 164 from the nonperinatal group with missing race and ethnicity and marital status data, leaving 1150 perinatal and 17 655 nonperinatal decedents. The mean (SD) age was 29.1 (7.4) years for perinatal decedents and 35.8 (10.8) years for nonperinatal decedents. In terms of race and ethnicity, 140 perinatal decedents (12.2%) and 1235 nonperinatal decedents (7.0%) were Black, 124 (10.8%) and 1275 (7.2%) were Hispanic, 782 (68.0%) and 14 123 (80.0%) were White, and 104 (9.0%) and 1022 (5.8%) were of other race or ethnicity. Table 1 provides the participant characteristics.

**Table 1.** Characteristics of Decedents (Nonperinatal, Pregnant, or Postpartum Within 1 Year), 2003 to 2021^a^

Characteristic	Perinatal vs nonperinatal decedents			Postpartum vs pregnant decedents		
	Perinatal (n = 1150)	Nonperinatal (reference) (n = 17 655)	Mean difference or OR (95% CI)^b^	Postpartum (n = 694)	Pregnant (reference) (n = 456)	Mean difference or OR (95% CI)^b^
Age, mean (SD)	29.1 (7.4)	35.8 (10.8)	−6.7 (−7.1 to 6.2)	28.8 (7.2)	29.6 (7.8)	−0.8 (−1.6 to 0.1)
Race and ethnicity						
Black	140 (12.2)	1235 (7.0)	2.05 (1.70-2.47)	75 (10.8)	65 (14.3)	0.77 (0.53-1.10)
Hispanic	124 (10. 8)	1275 (7.2)	1.77 (1.44-2.14)	83 (12.0)	41 (9.0)	1.34 (0.90-2.01)
White	782 (68.0)	14 123 (80.0)	[Reference]	470 (67.7)	312 (68.4)	[Reference]
Other race or ethnicity^c^	104 (9.0)	1022 (5.8)	1.84 (1.48-2.28)	66 (9.5)	38 (8.3)	1.15 (0.76-1.76)
Educational attainment						
Grade 12 or less	179 (15.6)	3316 (18.8)	[Reference]	110 (15.9)	69 (15.1)	[Reference]
High school diploma or GED	405 (35.2)	5535 (31.4)	1.36 (1.13-1.63)	241 (34.7)	164 (36.0)	0.92 (0.64-1.32)
Some college or college graduate or above	409 (35.6)	7233 (41.0)	1.05 (0.88-1.26)	254 (36.6)	155 (34.0)	1.03 (0.72-1.48)
Unknown^d^	157 (13.7)	1571 (8.9)	1.85 (1.48-2.31)	89 (12.8)	68 (14.9)	0.82 (0.53-1.27)
Marital status						
Single or never married	596 (51.8)	7530 (42.9)	[Reference]	355 (51.2)	241 (52.9)	[Reference]
Married or domestic partnership	386 (33.6)	5029 (28.5)	0.97 (0.85-1.11)	251 (36.2)	135 (29.6)	1.26 (0.97-1.65)
Divorced, separated, or widowed	168 (14.6)	5096 (28.9)	0.42 (0.35-0.50)	88 (12.7)	80 (17.5)	0.75 (0.53-1.05)
Other covariates						
Decedent injured at home	883 (76.8)	13 838 (78.4)	0.91 (0.79-1.05)	533 (76.8)	350 (76.8)	1.00 (0.76-1.33)
Autopsied (full or partial)	775 (67.4)	11 484 (65.1)	1.11 (0.98-1.26)	444 (64.0)	331 (72.6)	0.67 (0.52-0.87)
In labor force	420 (36.5)	6159 (34.9)	1.07 (0.95-1.22)	280 (40.4)	140 (30.7)	1.54 (1.12-1.96)
Availability of narrative on circumstances						
Circumstances known	1051 (91.4)	15 724 (89.1)	1.30 (1.06-1.61)	653 (94.1)	398 (87.3)	2.32 (1.53-3.53)
Mental health problem known	629 (54.8)	9696 (53.9)	0.99 (0.88-1.11)	433 (62.4)	196 (43.0)	2.20 (1.73-2.80)

Abbreviations: NVDRS, National Violent Death Reporting System; OR, odds ratio.

^a^ Unless specified otherwise, data are presented as the No. (%) of decedents. Decedents with missing race and ethnicity, age, and marital status (10 in the perinatal group and 164 in the nonperinatal group) were removed from the analytic sample.

^b^ For data presented as the No. (%) of decedents, values are expressed as OR (95% CI); for data presented as mean (SD), values are expressed as mean difference (95% CI).

^c^ Defined by the NVDRS and includes American Indian or Alaska Native, Asian, Native Hawaiian or Pacific Islander, and unspecified race or ethnicity.

^d^ Included in the statistical testing for educational attainment.

Of the 1150 perinatal decedents, 456 (39.6%) were pregnant at death, 203 (17.7%) were pregnant within 42 days of death, and 491 (42.7%) were pregnant 43 to 365 days before death, yielding 694 postpartum decedents. Of these 1150 decedents, 1032 met criteria for inclusion in the qualitative sample, including having a minimal narrative length of at least 34 characters. Of these 1032 decedents, 392 (38.0%) were pregnant at death, 187 (18.1%) were pregnant within 42 days of death, and 453 (43.9%) were pregnant between 43 and 365 days before death, yielding 640 postpartum decedents.

### Quantitative Findings

Compared with nonperinatal decedents, perinatal decedents were younger, were less likely to be White, had lower educational attainment, and were more likely to be married (Table 1). Among the perinatal sample, postpartum decedents were younger and were more likely to be married compared with pregnant decedents.

**Figure 2.**
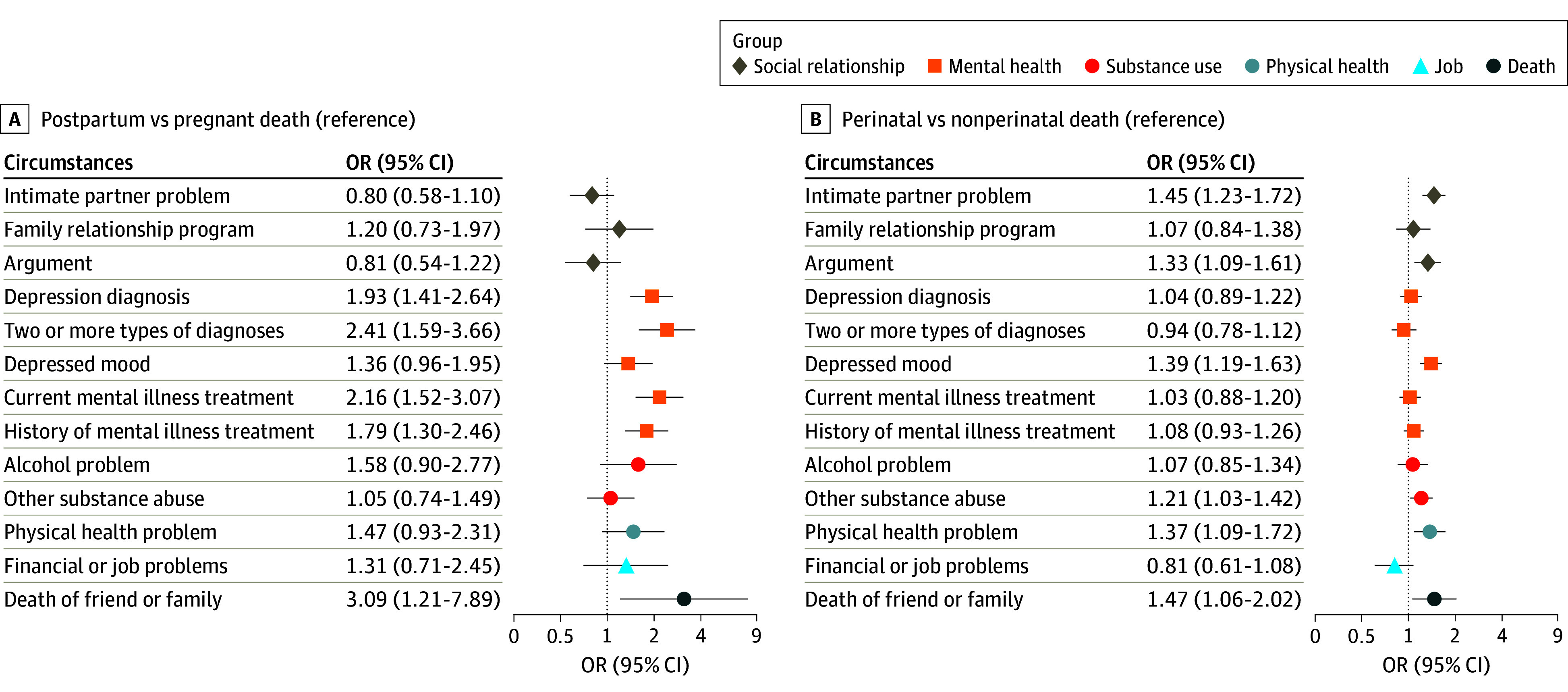
Odds Ratios (ORs) of Circumstances Contributing to Suicide A, Circumstances contributing to suicide among postpartum decedents relative to pregnant decedents (reference group). B, Circumstances contributing to suicide among perinatal decedents relative to nonperinatal decedents (reference group). Both forest logistic regression models and associated forest plots adjusted for age quartile, race and ethnicity, educational attainment, marital status, and other covariates (eg, injured at decedent’s home, autopsy, and in labor force).

Compared with pregnant decedents, postpartum decedents had higher odds of having had a depression diagnosis (conditional OR, 1.93 [95% CI, 1.41-2.64]), 2 or more mental illness diagnoses (OR, 2.41 [95% CI, 1.59-3.66]), depressed mood (OR, 2.03 [95% CI, 1.46-2.83]), current (OR, 2.16 [95% CI, 1.52-3.07]) or past (OR, 1.79 [95% CI, 1.30-2.46]) mental illness treatment, and death of a family member or friend (OR, 3.09 [95% CI, 1.21-7.89]) (Figure 2A). No significant differences for alcohol and substance use problems or relationship problems were observed.

Compared with nonperinatal decedents, perinatal decedents had higher odds of having had IPPs (OR, 1.45 [95% CI, 1.23-1.72]), recent argument (OR, 1.33 [95% CI, 1.09-1.61]), depressed mood (OR, 1.39 [95% CI, 1.19-1.63]), substance abuse or other abuse (OR, 1.21 [95% CI, 1.03-1.42]), physical health problems (OR, 1.37 [95% CI, 1.09-1.72]), and death of a family member or friend (OR, 1.47 [95% CI, 1.06-2.02]) (Figure 2B). eTable 1 in Supplement 1 presents data related to Figure 2.

In a post hoc sensitivity analyses, cause of death (suicide vs undetermined death) was not associated with pregnancy status (eTable 2 in Supplement 1). In the repeated regression analyses, after excluding undetermined deaths, key findings were substantially unchanged from the main analysis. Although the associations for 3 specific circumstances (eg, bereavement, substance abuse, and physical health) became insignificant, the direction of the association remained unchanged (eTable 3 in Supplement 1).

### Qualitative Findings

Table 2 summarizes key findings from 5 most common themes from the qualitative analysis, highlighting challenges related to mental health and substance use in the perinatal sample. Table 3 presents the frequency of all themes and subthemes for pregnant and postpartum groups. For example, 414 decedents (40.1%) had a diagnosis of depression. Substance use themes also included prescription drug use; 273 decedents (26.5%) used prescription drugs, which included cases of misuse or abuse. Qualitative analysis added to our understanding of relationship conflicts experienced by decedents. For example, IPPs occurred in 118 narratives (11.4%); 232 narratives (22.5%) described an argument or arguments within 48 hours prior to death. Qualitative analysis also highlighted themes uniquely relevant to decedents in the perinatal period. For example, we identified 105 decedents (10.2%) for whom having a miscarriage or stillbirth was a salient circumstance. Additionally, 76 decedents (7.4%) had or considered having an abortion within the past year.

**Table 2.** Summary of Qualitative Findings for the 5 Most Common Themes Among Pregnant and Postpartum Decedents

Major theme	Subthemes	Key findings	Exemplar narrative texts^a^
Intimate partner problems	Argument within 48 h Ongoing relationship problem Recent or past physical or verbal abuse Recent or past intimate partner problem Recent breakup or divorce	The theme and subthemes highlight the importance of timing and characterize the types of relationship conflicts experienced by perinatal decedents A total of 118 (11.4%) indicated an intimate partner problem and 127 (12.3%) a relationship problem A total of 232 (22.5%) described an argument or arguments with an intimate partner within the prior 48 h A total of 112 (10.9%) cited a recent relationship breakup, separation, or divorce Other features included timing of the conflict or breakup (ie, recent, past, or ongoing) and type of abuse (ie, verbal, physical, or sexual, if present)	“D was a recovering alcoholic and currently in a depressed mood. The D and her husband got into an argument the night prior over the D’s alcohol use.” “D had a recent breakup with an abusive boyfriend. The breakup was reportedly bad, and the D was upset.”
Mental health	Depressed mood Depression diagnosis Postpartum depression Multiple psychiatric comorbidities Any mental health treatment Recent treatment nonadherence Recent or past psychiatric hospitalization	The theme and subthemes highlight challenges around managing and treating ongoing (eg, previously diagnosed anxiety or depression) or recent (eg, postpartum depression) psychiatric conditions during the perinatal period Included diagnoses for depression (414 [40.1%]) or multiple psychiatric conditions (224 [21.7%]) A total of 152 (14.7%) stated that the decedent experienced depressed mood Postpartum depression noted for 128 decedents (12.4%) A total of 361 narratives (35.0%) indicated that the decedent had received some type of mental health treatment Other features included recent treatment nonadherence (62 [6.0%]) and history of psychiatric hospitalization (128 [12.4%])	“D was diagnosed with bipolar disorder and was taking medication. She had been depressed and had trouble sleeping. It was reported that she missed a recent appointment with her counselor.” “D was diagnosed with postpartum depression and insomnia. [D] also suffered from posttraumatic stress disorder from an abusive relationship with a previous partner. She was hospitalized for postpartum depression several months prior.”
Substance use	Illicit drug use, abuse, or both Use or abuse of prescription medication Alcohol use, abuse, or both Access to drugs via family member or acquaintance Drug overdose, unknown if intentional or unintentional	The theme and subthemes characterize decedent substance use and abuse A total of 382 decedents (37.0%) were described to use or abuse illicit drugs A total of 273 narratives (26.5%) indicated prescription drug use, which included cases of misuse or abuse For 14 decedents (1.3%), the narrative mentioned that the decedent obtained access to the drugs or medications from a family member or acquaintance A total of 260 decedents (25.2%) were known to use or abuse alcohol For 137 decedents (13.3%), the narrative mentioned that the decedent died of a drug overdose, but whether the overdose was intentional or unintentional was unclear	“D had a history of alcohol abuse and had previously been in rehab. D also had chronic pain from a herniated disk and was recently prescribed fentanyl patches. It was reported that D would abuse her prescription medications occasionally.” “D and her boyfriend had a history of illicit drug abuse. D also had recently begun abusing her prescription medications.”
Suicidal thoughts and behaviors	Recent or past suicidal thoughts or plans Recent or previous suicide attempts Suicide note found Suicide disclosure within 48 h	The theme and subthemes provide details about suicide risk behaviors, including timing of suicide intent disclosure Approximately one-quarter of decedents had a history of suicidal thoughts (309 [29.9%]) or suicide attempts (257 [24.9%]) A total of 167 decedents (16.2%) disclosed their suicide intent within 48 h of their death Narratives included text from suicide notes, if available, which provided additional contextual information	“D had a history of mental health problems and substance abuse. D had attempted suicide multiple times by overdose. D was recently given an involuntary mental health containment.” “The evening prior, the D threatened suicide to a friend. D asked her to take care of her children.”
Perinatal loss^b^	Recent or past death of a family member or friend Recent or past death of an infant child Had a miscarriage or stillbirth (may be from a prior pregnancy) Had or considered an abortion (may be from a prior pregnancy)	The theme and subthemes highlight perinatal loss (ie, bereavement, miscarriage or stillbirth, or abortion) as a circumstance Narratives described loss of a family member or friend (62 [6.0%]) or infant child (18 [1.7%]) within the past year A total of 105 decedents (10.2%) had a miscarriage or stillbirth within the past year A total of 76 decedents (7.4%) had or considered an abortion within the past year	“D had several previous pregnancies that ended in miscarriages. Recently, D had another miscarriage. D was referred to a psychiatrist, who reported that D was depressed about losing the baby.” “D had a history of depression, anxiety, and alcoholism. D had recently undergone an abortion. Since the abortion, D was reported to be very upset.”

Abbreviations: D, decedent; NVDRS, National Violent Death Reporting System.

^a^ Excerpts are composites from existing NVDRS narratives in our qualitative sample. They have been modified according to the 2023 NVDRS user guidelines to ensure confidentiality.^21^

^b^ Combines the results from bereavement and pregnancy termination (eAppendix 2 in Supplement 1) under the broader, representative theme of perinatal loss.

**Table 3.** Qualitative Themes of Precipitating Circumstances From Coroner, Medical Examiner, and Law Enforcement Narratives for Perinatal Decedents (Suicides and Undetermined Deaths)^a^

Theme^b^	All decedents (n = 1032)	Pregnant decedents (n = 392)	Postpartum decedents (n = 640)
Relationship conflicts			
Intimate partner			
Argument or fight within 48 h	232 (22.5)	100 (25.5)	132 (20.6)
Argument within 2 wk	45 (4.3)	16 (4.1)	29 (4.5)
Recent or past intimate partner problem	118 (11.4)	46 (11.7)	72 (11.3)
Ongoing relationship problem	127 (12.3)	39 (9.9)	88 (13.8)
Recent or past physical or verbal abuse	119 (11.5)	42 (10.7)	77 (12.0)
Recent or past sexual abuse	16 (1.6)	NA	NA
Recent breakup or divorce	112 (10.9)	36 (9.2)	76 (11.9)
Family relationship			
Argument or fight within 48 h	25 (2.4)	11 (2.8)	14 (2.2)
Argument within 2 wk	11 (1.1)	NA	NA
Recurring relationship problems	35 (3.4)	13 (3.3)	22 (3.4)
Recent or past physical or verbal abuse	13 (1.3)	NA	NA
Recent or past sexual abuse	15 (1.5)	6 (1.5)	9 (1.4)
Had other children	231 (22.4)	80 (20.4)	151 (23.4)
Community violence			
Sexual assault or abuse from friend, acquaintance, or stranger	25 (2.4)	13 (3.3)	12 (1.9)
Violent incident perpetrated by friend, acquaintance, or stranger	13 (1.3)	NA	NA
Mental health, substance use, and physical health history			
Mental health			
Depression diagnosis	414 (40.1)	139 (35.5)	275 (43.0)
Depressed mood	152 (14.7)	54 (13.8)	98 (15.3)
Postpartum depression	128 (12.4)	8 (2.0)	120 (18.8)
Multiple psychiatric comorbidities	224 (21.7)	62 (15.8)	162 (25.3)
Other or not specified mental health diagnosis	117 (11.3)	47 (12.0)	70 (10.9)
Recent or past psychiatric hospitalization	128 (12.4)	42 (10.7)	86 (13.4)
Any mental health treatment	361 (35.0)	110 (28.1)	251 (39.2)
Recent treatment nonadherence	62 (6.0)	19 (4.8)	43 (6.7)
Suicidal thoughts and behaviors			
Recent or past suicidal thoughts or plans	309 (29.9)	133 (33.9)	196 (30.6)
Suicide disclosure within 48 h	167 (16.2)	68 (17.3)	99 (15.5)
Suicide note found	238 (23.0)	87 (22.2)	151 (23.6)
Recent or previous suicide attempts	257 (24.9)	79 (20.1)	178 (27.8)
Physical health			
Presence of illness or physical health comorbidities	163 (15.8)	64 (16.3)	99 (15.5)
Recent or past hospitalization	64 (6.2)	21 (5.4)	43 (6.7)
Incident or crisis related to physical health within 48 h	15 (1.5)	7 (1.8)	8 (1.3)
Recent onset of chronic conditions or presence of terminal illness	41 (4.0)	20 (5.1)	21 (3.3)
Recent contact with medical or health care professional	61 (5.9)	21 (5.4)	40 (6.3)
Substance use			
Illicit drug use and abuse	382 (37.0)	152 (38.8)	230 (35.9)
Alcohol use or abuse	260 (25.2)	85 (21.7)	175 (27.3)
Use or abuse of prescription medication	273 (26.5)	99 (25.3)	174 (27.2)
Unknown if intentional or unintentional drug overdose	137 (13.3)	57 (14.5)	80 (12.5)
Access to illicit drug or prescription medication via family member or acquaintance	14 (1.3)	NA	NA
Contextual and social or environmental factors and other circumstances			
Socioeconomic disadvantage			
Recent loss of job or unemployed	50 (4.8)	19 (4.8)	31 (4.8)
Recent financial instability	41 (3.9)	17 (4.3)	24 (3.8)
Long-term financial instability	19 (1.8)	8 (2.0)	11 (1.7)
Housing			
Housing instability and moving or transitory living	88 (8.5)	33 (8.4)	55 (8.6)
Evicted or homeless	21 (2.0)	6 (1.5)	15 (2.3)
Interaction with justice or law enforcement			
Recent or past contact with law enforcement	101 (9.8)	44 (11.2)	57 (8.9)
Incarcerated at time of death	9 (0.9)	NA	NA
Past incarceration	25 (2.4)	7 (1.8)	18 (2.8)
Bereavement			
Recent or past death of family or friend	62 (6.0)	21 (5.4)	41 (6.4)
Recent or past death of infant child	18 (1.7)	NA	NA
Pregnancy termination			
Had or considered an abortion	76 (7.4)	21 (5.4)	55 (8.6)
Had miscarriage or stillbirth	105 (10.2)	12 (3.1)	93 (14.5)

Abbreviations: CDC, Centers for Disease Control and Prevention; NA, not available; NVDRS, National Violent Death Reporting System.

^a^ Unless specified otherwise, values are presented as the No. (%) of decedents.

^b^ Circumstances are not mutually exclusive. Cells with values less than 5 (denoted with NA) are suppressed to protect confidentiality, per the CDC data use agreement. Section 5 (“Circumstance Variables”) of the 2022 NVDRS coding manual is the most relevant to this study.^22^ The coding manual defines *crisis* as a current or acute event (within 2 weeks of death) that is indicated in one of the source reports (law enforcement, coroner, or medical examiner) to have contributed to the death.^22^ We used this timeframe in our subthemes to characterize and distinguish between recent and past events.

Overall, Table 2 demonstrates that qualitative analysis offers complementary findings to quantitative analysis. In general, qualitative themes were largely concordant with quantitative NVDRS circumstances. However, qualitative themes yielded richer and more nuanced insights for each domain. For instance, for IPPs, content analysis gave further insight into the timing of the conflict (ie, recent or past) or argument (ie, within 48 hours prior) and further characterized the type of relationship conflict (ie, abuse [verbal, physical, or sexual] or divorce or breakup) compared with using solely quantitative analysis. Similarly, for mental health themes, qualitative analysis identified detailed challenges beyond diagnosis, such as experiencing postpartum depression (including relapse) and treatment nonadherence. Insights from qualitative analysis added more detailed and contextual information to quantitative analysis, leading to more comprehensive understanding for each domain.

## Discussion

In this study, we investigated factors occurring with perinatal suicide to inform policy and prevention strategies. The US has the highest maternal mortality rate among developed countries, with striking racial and ethnic disparities,^4^ although nearly all of these maternal deaths remain preventable.^2^ Therefore, improved targeting of policy and prevention strategies has the potential to enhance outcomes and to begin to address this crisis.

Primary findings from this study suggest that perinatal decedents were more likely to have experienced circumstances such as IPPs, depressed mood, substance abuse, physical health, and recent bereavement relative to nonperinatal decedents. Qualitative analysis of narrative themes among perinatal decedents emphasized the importance of mental health. Conflict with an intimate partner represented another common finding in the qualitative analysis. Finally, quantitative and qualitative analyses illustrated information gaps bridged by using both types of NVDRS data and provided context for understanding how risk factors for suicide (eg, poor mental health) varied over the perinatal period.

Our qualitative analysis contained more nuanced information related to the perinatal circumstances than quantitative analysis alone. For example, qualitative analysis identified patients who experienced postpartum depression, miscarriage, and abortion. The NVDRS could integrate such qualitative codes into the coding manual to improve contextual information related to pregnancy. This finding highlights the potential benefit of qualitative analysis alongside quantitative analysis.

We observed some demographic differences in suicidality from other literature. Previous research found that non-Hispanic Black pregnant women had the highest prevalence of suicidal ideation, suicide attempts, and nonsuicidal intentional self-harm.^23^ Compared with nonperinatal decedents, perinatal decedents were younger, were less likely to be White, had lower educational attainment, and were more likely to be married. We did not observe any statistically significant differences between postpartum and pregnant decedents. These findings suggest that clinicians and policy makers should work to address the needs of these vulnerable populations.

Although perinatal decedents in this study were more likely to be married than nonperinatal decedents, single and never-married individuals represented more than half of perinatal decedents. Prior research found that unmarried pregnant women with lower educational attainment and lower income had a higher risk of intimate partner violence (IPV),^24^ a risk factor for perinatal suicide, indicating that these individuals may also require support and follow-up. High marital satisfaction during pregnancy may protect against suicidal ideation,^25^ and suicidal ideation occurs more commonly among unmarried pregnant women.^26^ Future research could explore interactions between risk factors for perinatal suicide and marital status.

In the present study, some circumstances overlapped between perinatal and postpartum decedents. For example, both were more likely than their comparison groups to experience mental health–related circumstances, such as depressed mood, substance abuse (perinatal), depression diagnosis, and current mental illness treatment or past mental illness treatment (post partum). In prior research, investigators found that mental health–related circumstances increased the risk of suicide or self-harm among perinatal individuals.^9,10,27,28^ Within this context, our study highlights the importance of perinatal mental health screening, including for suicidal ideation,^29^ and clinical follow-up.^30^

In this study, the aforementioned themes of substance and alcohol abuse represented 2 dominant qualitative circumstances and are an area for further research to better understand circumstances of perinatal decedents with these conditions. Women with substance or alcohol use disorders are also more likely to experience depression and IPV,^31^ presenting a complex picture of interrelated risk factors for suicide. Our finding that substance use disorders were more common among perinatal decedents suggests that improved care for perinatal individuals with substance use may reduce these deaths.

We observed a high frequency of IPPs for perinatal decedents in both the quantitative and qualitative results. Not all IPP instances represent IPV, because the NVDRS definition of IPPs includes normative conflicts, arguments, and divorce or separation, which may be nonviolent and thus may not constitute IPV.^22,32^ Prior research suggests that such non-IPV relationship factors nevertheless contribute to suicide risk.^22^ However, IPV also represents a subset of IPP cases.^33^ Prior research has also shown that perinatal suicide decedents were more likely to experience IPV,^34^ which may contribute to more than half of pregnancy-associated suicides.^35^ Indeed, IPV contributes to suicide risk across the lifespan.^32^ The co-occurrence of IPV with depression is also common.^36^ Validated IPV screening tools are available,^37^ yet more than half of individuals refuse IPV screening.^38^ Previous studies identified clinician-related barriers to IPV screening, including clinician discomfort, lack of knowledge, and time constraints.^39^ Policy makers and clinicians should target these barriers by supporting a comprehensive approach, such as the 2023 US National Plan to End Gender-Based Violence.^40^

Taken together, the findings of this study illuminate key areas for targeting policy and implementation of evidence-based interventions, with mental health, substance use, and IPV most relevant for mitigating perinatal suicide risk and enhancing maternal health outcomes. Screening often represents the first step of such interventions; however, in a recent systematic review that focused on screening for depression and suicide risk in the primary setting, the investigators found important evidence gaps related to understanding the benefits and harms of suicide screening.^41^ Researchers should urgently focus on these gaps, with the goal of better characterizing clinical implications, including suicide risk assessment and prevention strategies.

### Limitations

This study has multiple limitations related to underdocumentation, missingness, and misclassification. First, the NVDRS data depend on information available in source documents; subsequently, our results were subject to underdocumentation (ie, the absence of a circumstance does not mean that the circumstance was not present; rather, it was not documented). Prior research has shown that some decedents, such as racial and ethnic minority individuals and individuals with less educational attainment, are more likely to have missing narrative texts, which may affect the availability of qualitative data.^42^ Second, the NVDRS is a state-based database, and each state has independent reporting systems for violent deaths, resulting in bias in the assessment of circumstances.^12,43^ Moreover, the NVDRS has added new circumstance variables over time and it does not reassess old cases, resulting in additional missingness. Although we accounted for misclassifications of suicides as undetermined deaths by including these deaths in our analyses, we recognize that the NVDRS may have misclassified or undercounted additional deaths.

Our exposure (ie, pregnancy status) was subject to measurement error. Documentation of pregnancy status in early pregnancy, and of postpartum individuals who do not have custody of their children, may be limited.^34^ Prior literature suggests that some decedents may appear misclassified as pregnant on death certificates.^3,42^ Finally, although the NVDRS was recently expanded to all 50 states, its data availability varies based on the year that states opt in; as a result, the data may have limited generalizability.^34,44^

## Conclusions

In this cross-sectional observational study and qualitative analysis of 2003-2021 NVDRS data, we examined contextual and individual precipitating circumstances and risks associated with perinatal suicide. Our findings suggest that perinatal decedents experienced circumstances related to mental health, substance use, and IPPs. Perinatal decedents were also less likely to be White compared with other female decedents. Suicide death remains the leading cause of maternal mortality, and preventing maternal suicides represents an unmet public health need.^45^ Clinicians and policy makers should target these vulnerable populations by implementing existing evidence-based interventions and reducing stigma around reporting mental health conditions, substance use conditions, and IPPs in efforts to reduce maternal mortality by suicide.

## Notes

Supplement 1. **eAppendix 1.** Supplemental Details of Circumstance Variables for Quantitative Analysis, Adapted From the National Violent Death Reporting System Coding Manual (Version 6.0) **eMethods.** Matching Process for the Quantitative Analysis **eAppendix 2.** Qualitative Codebook With Definitions Annotators Used to Determine Themes and Subthemes to Assign Cases **eAppendix 3.** Narrative Analysis Process **eFigure.** Cross-Sectional Observational Study and Qualitative Analysis Design **eTable 1.** Quantitative Predictors From Figure 2 **eTable 2.** Post Hoc Sensitivity Analysis: Association Between Cause of Death (Suicide vs Undetermined Death) and Pregnancy Status **eTable 3.** Post Hoc Sensitivity Analysis: Associations Between Salient Circumstances and Suicide (Odds Ratios) Excluding Undetermined Deaths

Supplement 2.
Data Sharing Statement
