# Supplementary material for: Suicide Mortality During the Perinatal Period
Source: JAMA Netw Open. 2024 Jun 27;7(6):e2418887. doi: 10.1001/jamanetworkopen.2024.18887 (PMC11211960; doi:10.1001/jamanetworkopen.2024.18887)
Supplement: Supplement 1. — eAppendix 1. Supplemental Details of Circumstance Variables for Quantitative Analysis, Adapted From the National Violent Death Reporting System Coding Manual (Version 6.0) eMethods. Matching Process for the Quantitative Analysis eAppendix 2. Qualitative Codebook With Definitions Annotators Used to Determine Themes and Subthemes to Assign Cases eAppendix 3. Narrative Analysis Process eFigure. Cross-Sectional Observational Study and Qualitative Analysis Design eTable 1. Quantitative Predictors From Figure 2 eTable 2. Post Hoc Sensitivity Analysis: Association Between Cause of Death (Suicide vs Undetermined Death) and Pregnancy Status eTable 3. Post Hoc Sensitivity Analysis: Associations Between Salient Circumstances and Suicide (Odds Ratios) Excluding Undetermined Deaths [file jamanetwopen-e2418887-s001.pdf]

Supplementary Online Content

Zivin K, Zhong C, Rodríguez-Putnam A, et al. Suicide mortality during the perinatal period. *JAMA Netw Open*. 2024;7(6):e2418887.
doi:10.1001/jamanetworkopen.2024.18887

eAppendix 1. Details of Circumstance Variables for Quantitative Analysis, Adapted From the National Violent Death Reporting System Coding Manual (Version 6.0)

eMethods. Matching Process for the Quantitative Analysis

eAppendix 2. Qualitative Codebook With Definitions Annotators Used to Determine Themes and Subthemes to Assign Cases

eAppendix 3. Narrative Analysis Process

eFigure. Cross-Sectional Observational Study and Qualitative Analysis Design

eTable 1. Quantitative Predictors From Figure 2

eTable 2. Post Hoc Sensitivity Analysis: Association Between Cause of Death (Suicide vs Undetermined Death) and Pregnancy Status

eTable 3. Post Hoc Sensitivity Analysis: Associations Between Salient Circumstances and Suicide (Odds Ratios) Excluding Undetermined Deaths

This supplementary material has been provided by the authors to give readers additional information about their work.

© 2024 Zivin K et al. *JAMA Network Open.*

eAppendix 1. Details of Circumstance Variables for Quantitative Analysis, Adapted From the National Violent Death Reporting System Coding Manual (Version 6.0)

Circumstance Variable[a]	NVDRS Variable Name	Definition in NVDRS Coding Manual
Intimate Partner Problem	IntimatePartnerProblem_c	Problems with a current or former intimate partner appear to have contributed to the suicide or undetermined death, such as a divorce, break-up, argument, jealousy, conflict, or discord.
Family Relationship Problem	FamilyRelationship_c	Victim had relationship problems with a family member (other than an intimate partner) that appear to have contributed to the death.
Argument	Argument_c	An argument or conflict led to the victim's death.
Depression Diagnosis[b]	MentalHealthDiagnosis1,2,3,4_c	This variable indicates the nature of the victim's depression (the diagnosis)
Two or more diagnoses[b]	MentalHealthDiagnosis1,2,3,4_c	This variable indicates the nature of the victim's mental health problem (the diagnosis)
Depressed Mood	DepressedMood_c	Victim was perceived by self or others to be depressed at the time of the injury.
Current Mental Illness Treatment	MentalIllnessTreatmentCurrent_c	Currently in treatment for a mental health problem or substance abuse problem.
History of Mental Illness Treatment	HistoryMentalIllnessTreatmnt_c	History of ever being treated for a mental health or substance abuse problem.
Alcohol Problem	AlcoholProblem_c	Person has alcohol dependence or alcohol problem.
Other Substance Abuse	SubstanceAbuseOther_c	Person has a non-alcohol related substance abuse problem.
Physical Health Problem	PhysicalHealthProblem_c	Victim's physical health problem(s) appear to have contributed to the death
Financial or Job Problem[c]	FinancialProblem_c<br>JobProblem_c	Financial problems or Job problem(s) appear to have contributed to the death.
Death of a Family or Friend	DeathFriendOrFamilyOther_c	Death of a family member or friend due to something other than suicide appears to have contributed to the death.

National Violent Death Reporting System=NVDRS.

[a] All variables are binary variables, 0=No, not available, unknown, 1= Yes.

[b] NVDRS had four variables to record specific diagnosis of mental health problems. We created the variables of depression diagnosis and multiple psychiatric diagnoses separately according to the four variables.

[c] Due to the limited number of cases having "Yes" for financial problem and job problem, we combined them as Financial_Job problem in logistic regression.

© 2024 Zivin K et al. *JAMA Network Open.*

eMethods. Matching Process for the Quantitative Analysis

Matching process for comparison group 1: pregnant (control) vs. postpartum

We used propensity score matching to estimate the correlation between pregnancy status and 13 circumstances, accounting for confounding by the included covariates (race/ethnicity, education attainment, age group, state). We first attempted 1:1 nearest neighbor propensity score matching without replacement, in which a propensity score was estimated by logistic regression of the pregnancy status on covariates. This matching approach yielded poor balance, as the variance ratio did not increase, and the sample size decreased from 1,150 to 920, so we instead tried optimal full matching on the propensity score. Using optimal full matchings, we matched 694 of treated cases (postpartum group), and 456 of control cases (pregnant group) and generated 348 subclasses. After matching, the variance ratios indicated adequate balance (close to 1.0).

Matching process for comparison group 2: perinatal vs. non-perinatal

We used propensity score matching to estimate the correlation between whether the decedent died during the perinatal year and 13 circumstances, accounting for confounding by covariates (race/ethnicity, education attainment, age group, state). We first attempted 1:1 nearest neighbor propensity score matching without replacement and estimated a propensity score using logistic regression of the pregnancy status on covariates. This matching approach yielded poor balance, with a variance ratio estimated at 1.69, and the sample size decreased from 18,805 to 2,300. Instead, we tried optimal full matching on the propensity score. Using the optimal full matching, we matched 1,150 of treated cases (perinatal group) and 17,655 of control cases (non-perinatal group) and generated 1,118 subclasses. After matching, the variance ratios indicated adequate balance (close to 1.0).

© 2024 Zivin K et al. *JAMA Network Open.*

eAppendix 2. Qualitative Codebook With Definitions Annotators Used to Determine Themes and Subthemes to Assign Cases[a]

Domains (grey), themes (bold), and sub-themes	Detailed Sub-themes	Definition
**Relationship conflicts**		
**Intimate partner[b] problem**		
Argument or fight within 48 hours	--	V had a disagreement, debate, argument, or altercation with a current or former intimate partner within 48 hours prior to death.
Argument within two weeks	--	V had a disagreement, argument, or altercation with a current or former intimate partner that occurred within two weeks[c] prior to death.
Recent and/or past intimate partner problem	Recent intimate partner problem	V had problems with a current or former intimate partner, occurring within two weeks prior to death.
	Past intimate partner problem	V had problems with a current or former intimate partner, occurring within a year prior to death.
Ongoing relationship problem	--	V had recurring or persistent issues, difficulties, or disputes with an intimate partner
Recent and/or past physical or verbal abuse	Recent physical or verbal abuse	V experienced an incident or pattern of verbal abuse perpetrated by an intimate partner and or when an intimate partner hurt or tried to hurt a partner using physical force, occurring within two weeks prior to death.
	Past physical or verbal abuse	V experienced an incident or pattern of verbal abuse perpetrated by an intimate partner and or when an intimate partner hurt or tried to hurt a partner using physical force, occurring within one year prior to death.
History of sexual abuse	Recent sexual abuse	V experienced sexual abuse or assault perpetrated by a current or former intimate partner, occurring within two weeks prior to death.
	Past sexual abuse	V experienced sexual abuse or assault perpetrated by a current or former intimate partner, occurring within a year prior to death.
Recent breakup or divorce	Recent break-up	V and intimate partner recently ended the relationship, occurring within two weeks prior to death.
	Recent divorce	V and intimate partner were in the process of getting a divorce or had recently had a divorce within two weeks prior to death.
**Family relationship**		
Argument or fight within 48 hours	--	V had a disagreement, argument, or verbal altercation with a family member, other than an intimate partner, within 48 hours prior to death.
Argument within two weeks	--	V had a disagreement, argument, or verbal altercation with a family member, other than an intimate partner within two weeks prior to death.
Recent and/or past physical or verbal abuse	Recent physical or verbal abuse	V experienced an incident or pattern of verbal abuse perpetrated by a family member and/or when a family member hurt or tried to hurt V using physical force, occurring within two weeks prior to death.
	Past physical or verbal abuse	V experienced an incident or pattern of verbal abuse perpetrated by a family member and or when a family member hurt or tried to hurt V using physical force, occurring within a year prior to death.
History of sexual abuse	Recent sexual abuse	V experienced sexual abuse perpetrated by a family member, occurring within the past two weeks prior to death.
	Past sexual abuse	V experienced sexual abuse perpetrated by a family member, occurring within the past year prior to death.
Had other children	--	V had other children, outside their current or most recent pregnancy.
**Community violence**		
Sexual assault or abuse from friend, acquaintance, or stranger	--	V experienced sexual abuse perpetrated by a friend, acquaintance, or stranger.
Violent incident perpetrated by friend, acquaintance, or stranger	--	V experienced violence, other than sexual violence, perpetrated by a friend, acquaintance, or stranger.

© 2024 Zivin K et al. *JAMA Network Open.*

Domains (grey), themes (bold), and sub-themes	Detailed Sub-themes	Definition
**Mental health, substance use, and physical health history**		
**Mental health**		
Depression diagnosis	--	V received a depression diagnosis.
Depressed mood	--	V was depressed or continuously low, sad, down, etc.; an official diagnosis was not mentioned in the narrative.
Postpartum depression diagnosis	--	V was diagnosed with postpartum depression.
Multiple psychiatric comorbidities	--	V was diagnosed with a combination of two or more psychiatric disorders (e.g., depression, bipolar disorder, PTSD, dissociative identity disorder, anxiety, schizophrenia, OCD, among others).
Other or not specified mental health diagnosis	Other mental health diagnosis	V was diagnosed with a mental health issue other than depression or postpartum depression, and that was the sole mental health diagnosis stated.
	Not specified mental health diagnosis	V had mental health issues, but the narrative did not specify the diagnosis.
Recent and/or past psychiatric hospitalization	Recent hospitalization in psychiatric institution	V had been hospitalized in a psychiatric institution within two weeks prior to death.
	Past hospitalization in psychiatric institution	V had been hospitalized in a psychiatric institution up to a year prior to death.
Any mental health treatment	Had mental health counseling	V was attending or had attended sessions with a mental health counselor, therapist, or psychiatrist within one year prior to death.
	Had or was on psychiatric medication	V took or possessed prescription medication prescribed to treat psychiatric symptoms or condition(s).
Recent treatment nonadherence	Recent treatment nonadherence	V intentionally stopped regular treatments, counseling appointments or prescription medication within two weeks prior to death.
	Had counseling appointment bud did not attend	V intended and scheduled to attend a session with a mental health counselor, therapist, or psychiatrist within 48 hours before their death and didn't attend.
**Suicidal thoughts and behaviors**		
Recent and/or past suicidal thoughts or plans	Recent suicidal thoughts or plans	V expressed suicidal statements, thoughts, plans, or threats within two weeks and up to a year prior to death. This could have been communicated verbally or via talk, text, etc.
	Past suicidal thoughts or plans	V expressed suicidal statements, thoughts, plans, or threats within the past year prior to death. This could have been communicated verbally or via talk, text, etc.
Suicide disclosure within 48 hours	--	V explicitly disclosed suicidal thoughts or plans to others within 48 hours prior to death. This could have been communicated verbally or via talk, text, etc.
Suicide note found	--	Suicide note(s) written by the V were found by authorities, including paper, text messages, or email.
History of suicide attempt(s)	Recent suicide attempt(s)	V attempted suicide in the past within 48 hours to two weeks.
	Previous suicide attempt(s)	V attempted suicide in the past within the past year.
**Physical health**		
Presence of illness or physical health comorbidities	--	V was diagnosed with an illness that was not terminal or had other physical health comorbidities.
Recent and/or past hospitalization	Recent hospitalization	V was admitted to the hospital or had been recently discharged within two weeks prior to death.
	Past hospitalization	V was admitted to the hospital or had been recently discharged within the past year prior to death.
Incident or crisis related to physical health within 48 hours	--	V had an acute change or crisis related to physical health within 48 hours of death.

© 2024 Zivin K et al. *JAMA Network Open.*

Domains (grey), themes (bold), and sub-themes	Detailed Sub-themes	Definition
Recent onset of chronic conditions and/or presence of terminal illness	Recent worsening or onset of chronic conditions	V was diagnosed with a chronic (long-term) health condition recently or experienced worsened symptoms of health conditions/comorbidities.
	Terminal illness	V was aware that they were diagnosed with a terminal illness or condition.
Recent contact with medical or healthcare professional	--	V went to a medical appointment or was treated by a medical or healthcare professional within two weeks prior to death.
**Substance use**		
Illicit drug use and/or abuse	Illicit drug use	V used or abused illicit drugs, or their presence was detected in the toxicology report.
	Illicit drug abuse	V or others perceived that V had a problem with, or was addicted to drugs other than alcohol, or participated in a drug rehab program or treatment.
Alcohol use and/or abuse	Alcohol use	V consumed alcohol leading up to the fatal incident, and this was detected in the toxicology report.
	Alcohol abuse	V had alcohol dependence or was managing alcohol dependency (e.g., perceived by self or others, or it was mentioned that V had participated in an alcohol rehabilitation program).
Use or abuse of prescription medication	--	V received prescriptions or took medication(s) provided by a healthcare professional.
Unknown if intentional or unintentional drug overdose	--	The cause of death is undetermined and resulted from a drug overdose.
Access to illicit drug or prescription medication via family member or acquaintance	--	V obtained illicit drugs or unauthorized prescription medication(s) through interactions with family members or acquaintances, rather than through conventional medical channels or vendors.
**Contextual and social/environmental factors and other circumstances**		
**Socioeconomic disadvantage**		
Recent loss of job and/or unemployed	Recent loss of job	V lost their job (e.g., was terminated or furloughed) within two weeks prior to death.
	Unemployed	V was unemployed at time of death.
Recent financial instability	--	V experienced financial challenges that affected their ability to meet basic needs, within two weeks prior to death.
Long-term financial instability	--	V experienced long-term financial challenges that affected their ability to meet basic needs or pay aggregate debt within one year prior to death.
**Housing**		
Current housing instability and/or moving or transitory living	Current housing instability	V didn't have permanent or secure housing prior to death, or planned to move, or was staying in a temporary place (e.g., family's home, trailer, hotel, or motel).
	Moving or transitory living	V planned to move to a new state, and/ or was kicked-out from previous stable housing and stayed in a temporary place (e.g., family's home, trailer, hotel, or motel).
Recently evicted or homeless	Evicted or loss of home within 48 hours	V was just evicted or homeless within 48 hours prior to death.
	Recently evicted or homeless	V was evicted or homeless within two weeks up to a year prior to death.
**Interaction with justice or law enforcement**		
Recent and/or past contact with law enforcement	Recent contact with law enforcement	V had been questioned, issued a warrant, arrested, or any other type of contact with law enforcement within two weeks and up to a year prior to death.
	Past contact with law enforcement	V had been questioned, issued a warrant, arrested, or any other type of contact with law enforcement within a year prior to death.
Incarcerated at time of death	--	V was in jail or prison at the time of death.

© 2024 Zivin K et al. *JAMA Network Open.*

Domains (grey), themes (bold), and sub-themes	Detailed Sub-themes	Definition
Past incarceration	--	V was incarcerated in jail or prison within the past year prior to death.
**Bereavement**		
Death of family or friend	Recent death of family or friend	V experienced a death or deaths of a close family or friend within two weeks prior to death.
	Past death of family or friend	V experienced a death or deaths of a close family or friend between two weeks up to a year prior to death.
Death of an infant child	Recent death of an infant child	V's child of their most recent pregnancy died under the age of one year within two weeks prior to death.
	Past death of an infant child	V's child of their most recent pregnancy died under the age of one year between two weeks and a year prior to death.
**Pregnancy termination**		
Had or considered an abortion	Had abortion within 48 hours	V had an abortion of most recent pregnancy within 48 hours prior to death.
	Had a recent abortion	V had an abortion of most recent pregnancy within two weeks prior to death.
	Had a past abortion	V had an abortion of most recent pregnancy within a year.
	Scheduled or considered an abortion	V had planned, scheduled, or considered an abortion for most recent pregnancy.
Had a past or recent miscarriage or stillborn	Had a miscarriage or stillborn within 48 hours	V experienced a miscarriage or stillborn within 48 hours prior to death.
	Had a recent miscarriage or stillborn	V experienced a miscarriage of their most recent pregnancy within two weeks prior to death.
	Had a past miscarriage or stillborn	V experienced a miscarriage of a past pregnancy, within a period of one year prior to death.

C/ME=coroner and medical examiner; LE=law enforcement; National Violent Death Reporting System=NVDRS; PTSD=post-traumatic stress disorder; V=victim.

[a] This qualitative codebook builds on the circumstantial variables in the NVDRS, which means that some of the themes or sub-themes and their associated definitions have overlap with variables in the NVDRS quantitative data.

[b] "Intimate partner" refers to both current and former spouses and dating partners.

[c] NVDRS Coding Manual 6.0 (2022) defines "crisis" as a current or acute event (within 2 weeks of death) that is indicated in one of the source reports (LE or C/ME) to have contributed to the death. We are using this timeframe in our sub-themes to characterize and distinguish between recent and past events.

© 2024 Zivin K et al. *JAMA Network Open.*

eAppendix 3. Narrative Analysis Process

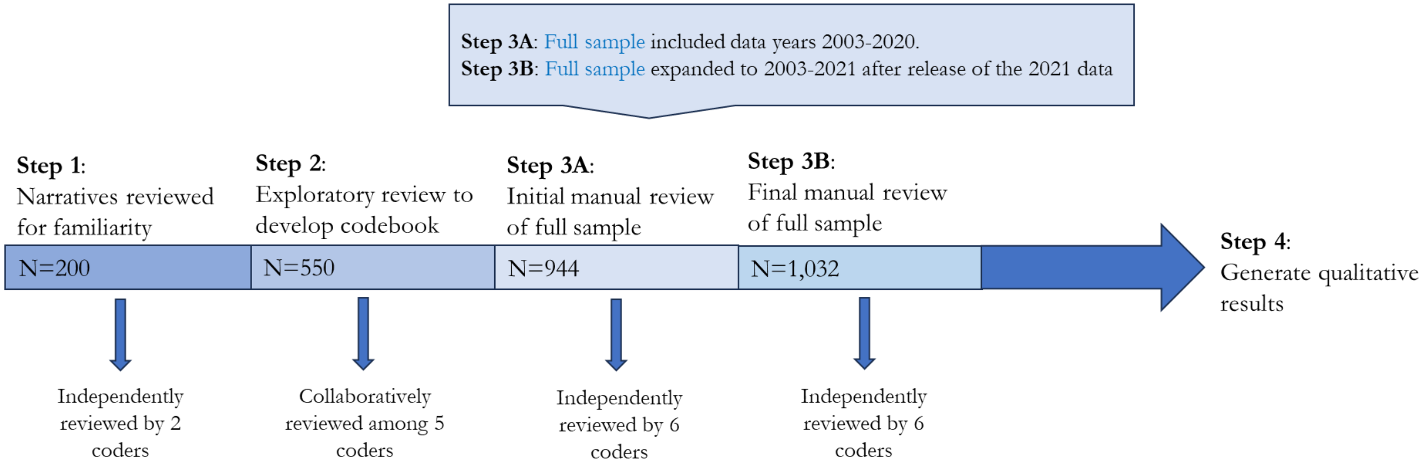

- **Step 1.** Two independent annotators conducted an exploratory manual review of an initial subset (n=200) to gain familiarity with the data and inductively generate emergent themes
- **Step 2.** Five independent annotators conducted a second exploratory manual review to co-build/construct codebook with detailed/specific themes related to the perinatal sample using a subset of this sample (n=550)
  - Based on the grounded theory and thematic analysis, we developed open-coding procedures that accounted for codes in the NVDRS Coding Manual (v.6.0), but we did limit the analyses to these codes to identify overarching themes describing the incident. Annotators met to discuss discrepancies and consensus coding procedures. Ultimately, the codebook has 12 major themes (representing 48 sub-themes), organized within 3 overarching domains: a) relationship conflicts, b) mental health, substance use, and physical health history, c) contextual and social/environmental factors and other circumstances.
- **Step 3A.** Six independent annotators completed a first round of annotations with 2003-2020 NVDRS data (n=944). The codebook was updated by tweaking minor changes, eliminating redundancy, and combining codes to produce the final product (Appendix 3).
- **Step 3B.** Six independent annotators completed a second round of annotations with 2003-2021 NVDRS data (n=1,032) using the updated and refined codebook. To assess inter-annotator agreement, Krippendorff's Alpha reliability coefficient was calculated using "kripp.alpha" function from "irr" package in R, as an aggregate value across 12 main themes. Inter-annotator was strong (0.84), which represents a high level of agreement among annotators, validating consistency and reliability of our coding process.
- **Step 4.** Generated qualitative results of most frequent themes relevant to the perinatal sample.

© 2024 Zivin K et al. *JAMA Network Open.*

eFigure. Cross-Sectional Observational Study and Qualitative Analysis Design

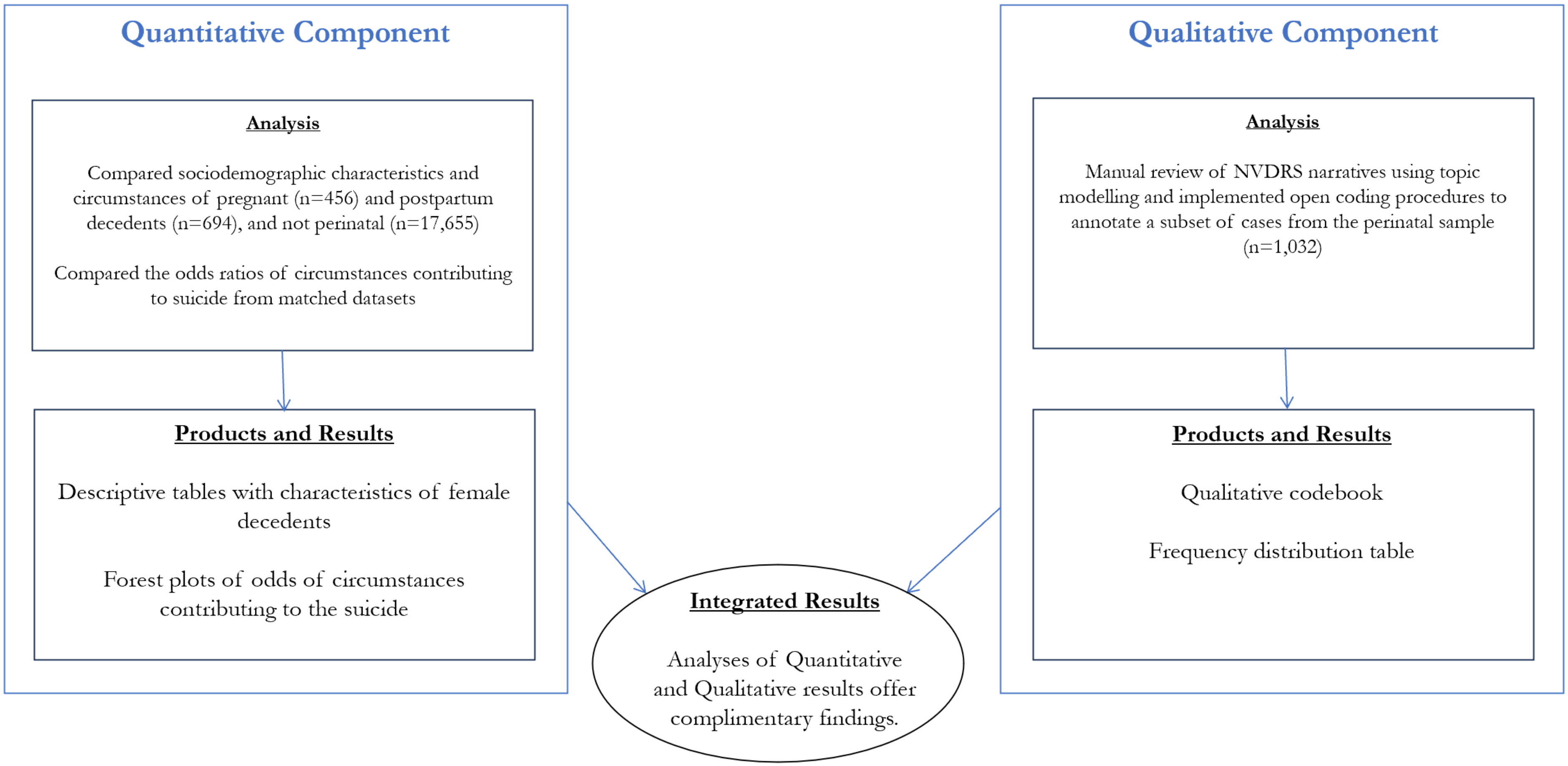

© 2024 Zivin K et al. *JAMA Network Open.*

eTable 1. Quantitative Predictors From Figure 2

Variable (N, %)	Non-perinatal (N=17,655)	Perinatal (N=1,150)				Pregnant (N=456)	Postpartum (N=694)			
**Circumstances**			**OR**	**95%CI**	**p-value**			**OR**	**95%CI**	**p-value**
**Social relationship**										
Intimate partner problem	**4,432 (25.10%)**	**436 (37.91%)**	**1.45**	**(1.23, 1.72)**	**<0.001**	171 (37.50%)	265 (38.18%)	0.80	(0.58, 1.10)	0.17
Family relationship problem	1,584 (8.97%)	106 (9.22%)	1.07	(0.84, 1.38)	0.58	37 (8.11%)	69 (9.94%)	1.20	(0.73, 1.97)	0.48
Argument	**2,305 (13.06%)**	**246 (21.39%)**	**1.33**	**(1.09, 1.61)**	**<0.001**	98 (21.49%)	148 (21.33%)	0.81	(0.54, 1.22)	0.31
**Mental health [a]**										
Depression diagnosis	7,057 (39.97%)	451 (39.22%)	1.04	(0.89, 1.22)	0.61	134 (29.39%)	317 (45.68%)	**1.93**	**(1.41, 2.64)**	**<0.001**
Two or more diagnoses	3,878 (21.97%)	245 (21.30%)	0.94	(0.78, 1.12)	0.48	65 (14.25%)	180 (25.94%)	**2.41**	**(1.59, 3.66)**	**<0.001**
Depressed mood	**5,467 (30.97%)**	**408 (35.48%)**	**1.39**	**(1.19, 1.63)**	**<0.001**	120 (26.32%)	288 (41.50%)	1.36	(0.96, 1.95)	0.09
Current treatment for mental illness	6,632 (37.56%)	395 (34.35%)	1.03	(0.88, 1.20)	0.74	119 (26.10%)	276 (39.77%)	**2.16**	**(1.52, 3.07)**	**<0.001**
History of treatment for mental illness	8,103 (45.90%)	508 (44.17%)	1.08	(0.93, 1.26)	0.29	165 (36.18%)	343 (49.42%)	**1.79**	**(1.30, 2.46)**	**<0.001**
**Substance use**										
Alcohol problem	2,656 (15.04%)	152 (13.22%)	1.07	(0.85, 1.34)	0.56	50 (10.96%)	102 (14.07%)	1.58	(0.90, 2.77)	0.11
Substance abuse, other abuse	**4,769 (27.01%)**	**348 (30.26%)**	**1.21**	**(1.03, 1.42)**	**0.02**	136 (29.82%)	212 (30.55%)	1.05	(0.74, 1.49)	0.77
**Health domain**										
Physical problem	**2,656 (15.04%)**	**137 (11.91%)**	**1.37**	**(1.09, 1.72)**	**0.01**	50 (10.96%)	87 (12.54%)	1.47	(0.93, 2.31)	0.10
**Job domain [b]**										
Financial or job problem	1,881 (10.65%)	83 (7.22%)	0.81	(0.61, 1.08)	0.15	32 (7.02%)	51 (7.35%)	1.31	(0.71, 2.45)	0.39
**Death**										
Death of family member or friend	**898 (5.09%)**	**65 (5.65%)**	**1.47**	**(1.06, 2.02)**	**0.02**	11 (2.41%)	54 (7.78 %)	**3.09**	**(1.21, 7.89)**	**0.02**

[a] Depressed mood (NVDRS variable DepressedMood_c) is coded 1 if 'Yes' and 0 otherwise. Depression diagnosis (NVDRS variable MentalHealthDiagnosis_c1,2,3,4) is coded 1 if 'Yes'. These two variables are not mutually exclusive. More information of the circumstances variables appears in Appendix 2).

[b] According to the NVDRS Coding Manual, "Financial/Job problem" is a circumstance that is thought to contribute to suicide. It is different from the "in labor force" variable in the Table 1, which is a characteristic of the decedent.

© 2024 Zivin K et al. *JAMA Network Open.*

eTable 2. Post Hoc Sensitivity Analysis: Association Between Cause of Death (Suicide vs Undetermined Death) and Pregnancy Status

	Suicide	Undetermined Death	Chi-square (p-value)	OR (95% CI)
**Comparison group 1**				
Perinatal	919 (6.07%)	231 (6.32%)	0.33 (0.56)	0.96 (0.82, 1.11)
Non-perinatal	14,232 (93.93%)	3,423 (93.68%)		
**Comparison group 2**				
Pregnant	356 (38.74%)	100 (43.29%)	1.60 (0.21)	0.83 (0.62 1.11)
Postpartum	563 (61.26%)	131 (56.71%)		

© 2024 Zivin K et al. *JAMA Network Open.*

eTable 3. Post Hoc Sensitivity Analysis: Associations Between Salient Circumstances and Suicide (Odds Ratios) Excluding Undetermined Deaths

Variable (N,%)	Non-perinatal (N=14,232)	Perinatal (N=919)				Pregnant (N=356)	Postpartum (N=563)			
**Circumstances**			OR	95%CI	p-value			OR	95%CI	p-value
**Social relationship**										
Intimate partner problem	4,093 (28.76%)	402 (43.74%)	**1.62**	**(1.33, 1.96)**	**<0.001**	160 (44.94%)	242 (42.98%)	0.84	(0.56, 1.24)	0.38
Family relationship problem	1,462 (10.27%)	95 (10.34%)	1.35	(1.00, 1.82)	0.05	34 (9.55%)	61 (10.83%)	1.48	(0.83, 2.64)	0.19
Argument	2,133 (14.99%)	225 (24.48%)	**1.53**	**(1.23, 1.90)**	**<0.001**	90 (25.28%)	135 (23.98%)	0.79	(0.51, 1.21)	0.27
**Mental health**										
Depression diagnosis	6,161 (43.29%)	389 (42.33%)	1.11	(0.93, 1.34)	0.24	119 (33.43%)	270 (57.96%)	**1.60**	**(1.10, 2.34)**	**0.01**
Two or more diagnoses	3,295 (23.15%)	203 (22.09%)	1.06	(0.86, 1.32)	0.56	56 (15.73%)	157 (26.11%)	**1.91**	**(1.22, 2.99)**	**<0.001**
Depressed mood	5,014 (35.23%)	383 (41.68%)	**1.33**	**(1.10, 1.61)**	**<0.001**	115 (32.20%)	268 (47.60%)	**1.49**	**(1.01, 2.19)**	**0.04**
Current treatment for mental illness	5,495 (38.61%)	327 (35.58%)	0.99	(0.82, 1.20)	0.92	101 (28.37%)	226 (40.14%)	**1.73**	**(1.10, 2.72)**	**0.02**
History of treatment for mental illness	6,763 (47.52%)	423 (46.03%)	1.13	(0.94, 1.37)	0.19	139 (39.04%)	284 (50.44%)	**1.58**	**(1.04, 2.40)**	**0.03**
**Substance use**										
Alcohol problem	2,138 (15.02%)	120 (13.06%)	0.98	(0.74, 1.29)	0.86	35 (9.83%)	85 (15.10%)	1.37	(0.76, 2.48)	0.29
Substance abuse, other abuse	2,946 (20.70%)	204 (22.20%)	1.09	(0.87, 1.36)	0.46	74 (20.79%)	130 (23.09%)	1.18	(0.76, 1.82)	0.46
**Health domain**										
Physical problem	1,914 (13.45%)	108 (11.75%)	1.26	(0.93, 1.71)	0.13	38 (10.67%)	70 (12.43%)	1.11	(0.63, 1.93)	0.72
**Job domain**										
Financial or job problem	1,734 (12.18%)	78 (8.49%)	0.73	(0.53, 1.02)	0.06	32 (8.99%)	46 (8.17%)	1.23	(0.69, 2.21)	0.48
**Death**										
Death of family member or friend	781 (5.49%)	57 (6.20%)	1.31	(0.89, 1.92)	0.17	11 (3.09%)	46 (8.17%)	1.53	(0.67, 3.49)	0.31

Significant results **bolded**

© 2024 Zivin K et al. *JAMA Network Open.*
