# Supplementary material for: Suicide Mortality During the Perinatal Period
Source: JAMA Netw Open. 2024 Jun 27;7(6):e2418887. doi: 10.1001/jamanetworkopen.2024.18887 (PMC11211960; doi:10.1001/jamanetworkopen.2024.18887)
Supplement: Supplement 2. — Data Sharing Statement [file jamanetwopen-e2418887-s002.pdf]

Data Sharing Statement

Zivin. Suicide Mortality During the Perinatal Period. *JAMA Netw Open*. Published June 27, 2024. doi:10.1001/jamanetworkopen.2024.18887

Data
Data available: No
